# Association of Omeprazole‐Related Myopathy With Drug–Drug and Drug–Gene Interactions Involving CYP2C19 and CYP3A4: A Nested Case–Control Study

**DOI:** 10.1002/phar.70058

**Published:** 2025-09-08

**Authors:** Eugene Jeong, Aditi Shendre, Yu Su, Xingyi Guo, Lang Li, You Chen

**Affiliations:** ^1^ Division of Epidemiology, Department of Medicine, Vanderbilt Epidemiology Center, Vanderbilt‐Ingram Cancer Center Vanderbilt University School of Medicine Nashville Tennessee USA; ^2^ Department of Biomedical Informatics, College of Medicine The Ohio State University Columbus Ohio USA; ^3^ Department of Computer Science and Engineering, College of Engineering The Ohio State University Columbus Ohio USA; ^4^ Department of Biomedical Informatics, School of Medicine Vanderbilt University Medical Center Nashville Tennessee USA; ^5^ Department of Computer Science, School of Engineering Vanderbilt University Nashville Tennessee USA

**Keywords:** biobank, drug interactions, EHR, omeprazole, pharmacogenomics, pharmacokinetics, translational science

## Abstract

**Background:**

Omeprazole, a widely used proton pump inhibitor, has been associated with rare but serious adverse events such as myopathy. Previous research suggests that concurrent use of omeprazole with fluconazole, a potent cytochrome P450 (CYP) 2C19/3A4 inhibitor, may increase the risk of myopathy. However, the contribution of genetic polymorphisms in CYP enzymes remains unclear.

**Aims:**

This study leveraged electronic health record (EHR) and biobank data to validate an interaction between omeprazole and fluconazole and to explore drug–gene interactions (DGIs) between omeprazole and polymorphisms in CYP enzymes.

**Materials and Methods:**

A nested case–control design with incidence‐density matching was used. Cases were defined as patients who developed myopathy during ongoing omeprazole therapy. For each case, up to four controls were selected from patients who had not developed myopathy by the time the case was diagnosed. Conditional logistic regression models, adjusting for relevant covariates, evaluated (i) the association between concomitant fluconazole use and myopathy and (ii) genotype‐stratified myopathy risk.

**Results:**

Among 902 cases and 3608 controls, the combined use of omeprazole and fluconazole was linked to an increased risk of myopathy (adjusted odds ratio [AOR] = 1.75, 95% confidence interval [CI]: 1.17–2.63, *p* = 0.007). In the DGI analysis, which included 862 cases and 3448 controls, individuals classified as CYP2C19 poor metabolizers paired with CYP3A4 extensive metabolizers showed a significantly higher myopathy risk (AOR = 1.62, 95% CI: 1.03–2.55, *p* = 0.036); those with CYP2C19 poor metabolizer/CYP3A4 intermediate metabolizer had an even greater risk (AOR = 4.77, 95% CI: 1.74–13.1, *p* = 0.002).

**Discussion:**

These findings not only confirm previously reported drug–drug interactions (DDIs) between omeprazole and fluconazole but also reveal the emerging clinical implications of DGIs.

**Conclusion:**

By integrating EHR and genetic data, the study showcases how informatics tools can translate DDI findings into DGI hypotheses, effectively bridging genetic insights and clinical outcomes.

## 1 Introduction

Omeprazole, a widely prescribed proton pump inhibitor (PPI), is extensively used to treat gastroesophageal reflux disease (GERD), peptic ulcers, and Zollinger‐Ellison syndrome due to its potent inhibition of gastric acid secretion [1]. As one of the most prescribed drugs in the United States, with over 52 million prescriptions written in 2019 [2], omeprazole is generally considered safe and well‐tolerated by most patients, with common side effects being mild, such as headaches, nausea, diarrhea, abdominal pain, and dizziness [3]. Nonetheless, rare yet serious adverse drug events (ADEs) have been documented, such as myopathy [4, 5]—a muscle disorder characterized by weakness that can impair quality of life [6]. In severe cases, it can progress to rhabdomyolysis, a condition that can lead to renal failure and life‐threatening complications [7].

Although the mechanistic link between omeprazole and myopathy remains incompletely understood, emerging evidence suggests three potential pathways. First, long‐term omeprazole use is strongly associated with hypomagnesemia due to impaired intestinal magnesium absorption [8], which disrupts neuromuscular function and calcium homeostasis, predisposing patients to muscle weakness, cramps, and myopathy [8]. Second, autoimmune mechanisms have been reported, where cross‐reactive antibodies targeting muscle‐specific antigens such as H^+^/K^+^‐ATPase may trigger acute‐onset myopathy in genetically susceptible individuals [9, 10]. Third, drug–drug interactions (DDIs)—particularly those involving cytochrome P450 (CYP450) enzymes—represent a modifiable and clinically significant risk [11]. Omeprazole is metabolized primarily by CYP2C19 and, to a lesser extent, by CYP3A4 [12], and concomitant use with CYP2C19 and CYP3A4 inhibitors such as fluconazole can elevate omeprazole plasma concentrations, leading to drug accumulation and direct myocyte injury [5, 12, 13].

Although omeprazole and fluconazole are not typically prescribed together for a single medical condition, there are clinical scenarios where a patient may require both concurrently. For example, patients with GERD may develop fungal infections, such as esophageal candidiasis, especially if they are immunocompromised. In such cases, omeprazole is used to manage GERD symptoms, while fluconazole treats the fungal infection [14, 15]. Due to its inhibitory effect on CYP2C19 and CYP3A4 [16, 17], fluconazole markedly reduces the hepatic clearance of omeprazole, leading to increased plasma concentrations [12, 13]. A pharmacokinetic study in healthy subjects demonstrated that administering fluconazole significantly decreased omeprazole clearance by approximately 40%–60%, highlighting the clinical importance of this interaction [12]. Additionally, a previous study that used both the United States Food and Drug Administration (FDA) Adverse Event Reporting System (FAERS) database and Indiana Network Patient Care data tested and validated a significant association between the co‐administration of omeprazole with fluconazole and an increased risk of myopathy [5]. However, these studies were limited by the lack of detailed consideration of patient‐specific risk factors, particularly CYP450 phenotypes, which play a critical role in drug metabolism.

In addition to DDIs, genetic polymorphisms in CYP2C19 and CYP3A4 may contribute to interindividual variability in omeprazole metabolism through drug–gene interactions (DGIs), thereby influencing both therapeutic outcomes and the risk of ADEs [18, 19]. CYP2C19 exhibits considerable genetic variability, with alleles that confer poor (PM), intermediate (IM), extensive (EM), and ultra‐rapid (UM) metabolizer phenotypes [20]. PMs, prevalent in approximately 15%–20% of Asian populations and around 3% of Caucasians, demonstrate higher systemic exposure to omeprazole, prompting the FDA to recommend dose adjustments in certain populations [21, 22, 23]. However, the drug label only notes that individuals with a CYP2C19 PM phenotype may experience adverse effects, without specifying which serious ADEs may occur.

The Synthetic Derivative (SD) [24] and BioVU biobank [25] at Vanderbilt University Medical Center (VUMC) represent significant advances in large‐scale pharmacogenomic (PG) research by combining de‐identified electronic health records (EHRs) with genetic data. The SD contains a large collection of de‐identified EHRs, and BioVU connects these records to DNA samples, resulting in a valuable resource for studying genetic factors that influence drug response and ADEs. This integrated platform has enabled numerous landmark PG studies, including the PREDICT project, which used preemptive genotyping to personalize drug therapy [26] and discovered genetic variants associated with warfarin dosing [27] and statin‐induced myopathy [28], demonstrating the potential of combining genomic data with EHRs to advance personalized medicine. Therefore, in this article, using SD and BioVU as the primary data source, we first performed an independent validation study on the association between the omeprazole–fluconazole interaction and the risk of myopathy. Then, we further investigated the impact of CYP2C19 and CYP3A4 polymorphisms on the incidence of myopathy in patients treated with omeprazole. By considering both DDIs and DGIs, our work aims to enhance the understanding of omeprazole‐related myopathy and contribute to personalized medicine approaches in PPI therapy.

## 2 Methods

### 2.1 Data Sources

For both DDI and DGI analyses, we utilized the SD database [24] and the BioVU database [25]. The SD database is a de‐identified EHR repository structured in the Observational Medical Outcomes Partnership Common Data Model (OMOP CDM) [29], facilitating standardized analyses across diverse clinical datasets, including demographics, diagnoses, medications, laboratory results, clinical notes, and procedural information organized into OMOP's systematic tables. We used the SD database updated through June 2025. Complementing this, BioVU is Vanderbilt's biorepository [25] that links DNA samples to the SD database through patient IDs, enabling researchers to combine genomic data with clinical phenotypes derived from EHRs for comprehensive genotype–phenotype studies in precision medicine. Through the Alliance for Genomic Discovery (AGD)—a collaboration between VUMC, Nashville Biosciences, and industry partners such as Illumina—BioVU currently houses whole‐genome sequencing (WGS) data from approximately 250,000 patients in the SD database. WGS samples were prepared using Illumina's DNA PCR‐Free Prep method (Illumina Inc., San Diego, CA, USA), which employs bead‐linked transposomes to simultaneously fragment genomic DNA and ligate sequencing adaptors. Approximately 500 ng of DNA per sample was quantified using Lunatic UV/Vis plate readers (Unchained Labs, Pleasanton, CA, USA) and processed in a fully automated workflow on Hamilton STAR NGS liquid handlers (Hamilton Company, Reno, NV, USA) with Illumina‐validated scripts. Each sample was indexed with dual indexes, stored at −20°C, pooled, quantified with the Qubit single‐stranded DNA (ssDNA) assay (Thermo Fisher Scientific, Waltham, MA, USA), and sequenced on Illumina NovaSeq 6000 instruments (Illumina Inc., San Diego, CA, USA) using paired‐end 151 × 10 × 10 × 151 cycle. Quality control (QC) involved confirming sample volume, DNA concentration, and A260/A280 ratios (1.8–2.0), alongside genetic sex checks at intake, followed by post‐sequencing assessments of guanine–cytosine (GC) content, insert size distribution, correct read pairing, and ensuring ≥ 85% of bases reached Q30 quality. Base calling was performed in real‐time on each sequencer via NovaSeq Control Software version 1.8.0 (Illumina Inc., San Diego, CA, USA), and alignment to GRCh38 was completed in real‐time using an in‐house pipeline on the DRAGEN server (Illumina Inc., San Diego, CA, USA). Rigorous QC thereafter included evaluating GC content, library insert size, and proper read pairing. Libraries underwent at least two sequencing rounds to ensure ≥ 30× mean autosomal coverage in ≥ 95% of samples, with ≥ 95% of autosome regions covered at ≥ 15×. Samples failing these criteria were subjected to additional top‐up sequencing using the NovaSeq‐XP workflow DRAGEN server (Illumina Inc., San Diego, CA, USA). Final quality metrics required contamination ≤ 1%, genetic sex verification matching reported data, ≥ 90% properly paired reads, genotype discordance < 2%, and a sequencing yield of ≥ 80 Gb of high‐quality bases. All sequencing metadata and final CRAM files were managed and recorded via Illumina Connected Analytics.

The study received approval from the Institutional Review Board (IRB) at VUMC, under approval identifier #221125.

### 2.2 Exposure: Omeprazole

**FIGURE 1:**
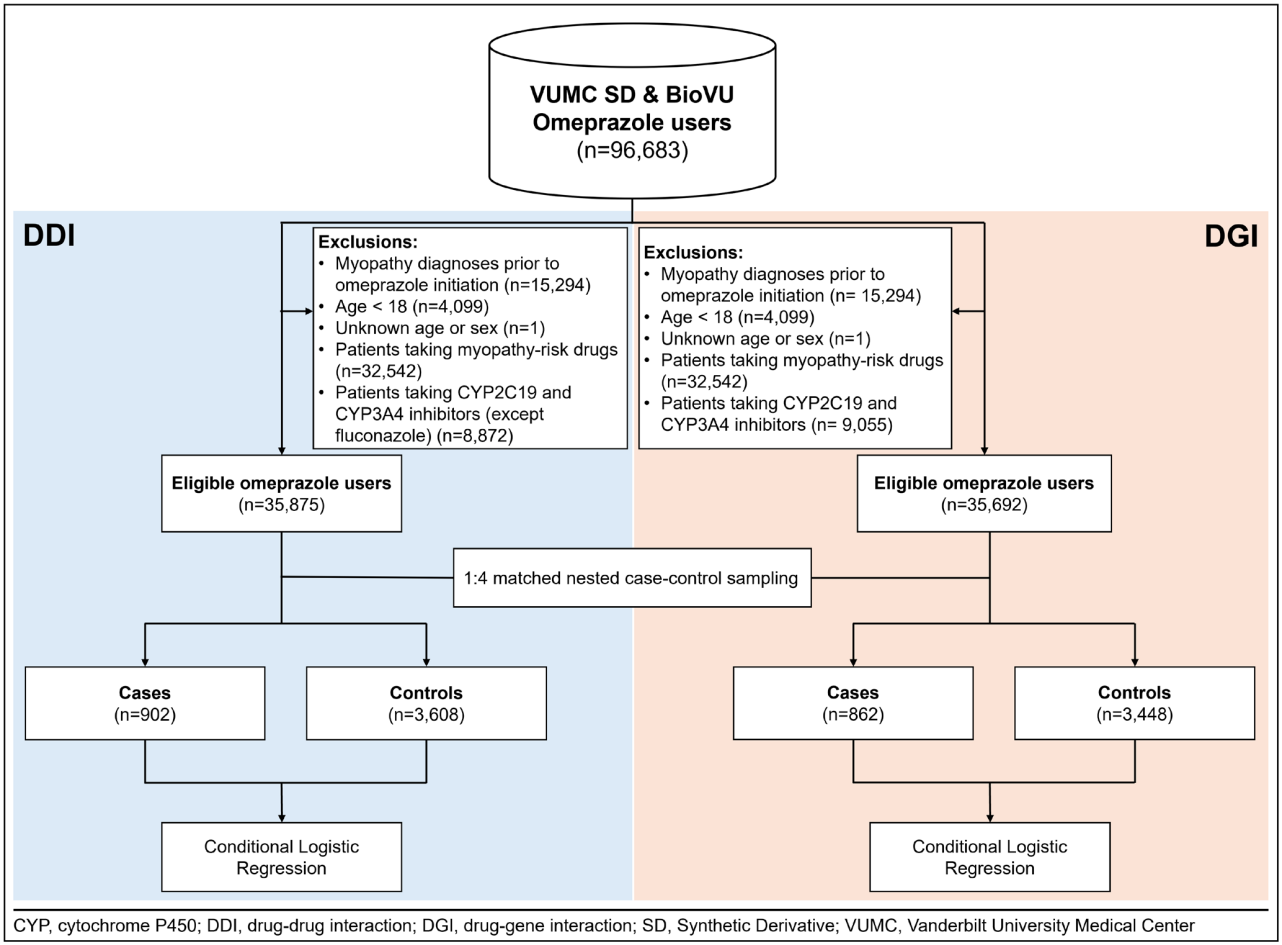
DDI and DGI study flow.

The study included all new users of omeprazole. Prescription records were used to identify omeprazole use based on the RxNorm classification (RxCUI: 7646). Any drug product that is a descendant or lower‐level concept of the omeprazole ingredient, meaning all drugs containing the ingredient omeprazole, was included in this analysis. There were 96,683 omeprazole users in the SD database who also had genetic data available in the BioVU data (Figure 1).

We excluded patients who met any of the following criteria: (i) younger than 18 years, (ii) missing information on age and sex, (iii) taking drugs that act as inhibitors of the CYP2C19 and CYP3A4 within 60 days before omeprazole initiation or within omeprazole exposure post‐initiation (note that fluconazole was not excluded in the DDI analysis, but it was excluded in the DGI analysis), (iv) taking drugs known to increase the risk of myopathies within 60 days before omeprazole initiation or within omeprazole exposure post‐initiation to avoid confounding from other drug‐induced myopathies, or (v) having any documented myopathy diagnosis before omeprazole initiation (Figure 1).

CYP2C19 and CYP3A4 inhibitors were identified using the FDA's drug development and interaction tables [16] and the Drug Interactions Flockhart table [17]. Both sources were used to ensure comprehensive identification of inhibitors, as each may include different drugs based on their distinct evidence bases, and both have been widely referenced in previous studies [30, 31, 32, 33]. The FDA tables are based on regulatory submissions and integrate both clinical and preclinical evidence reviewed during the drug approval process, whereas the Flockhart Table is primarily curated from published scientific literature. Both resources categorize inhibitors as strong, moderate, or weak based on the extent to which they increase the area under the curve (AUC) of sensitive substrates. Inhibitors with unknown strength were excluded. When discrepancies in inhibitor classification were noted between the two sources, the FDA table was used, as it represents a higher standard of evidence than the literature‐based Flockhart Table. Comprehensive lists of CYP2C19 and CYP3A4 inhibitors are provided in Table S1.

Drugs associated with an increased risk of myopathy were identified from the OnSIDES database [34], which comprises over 7.1 million drug–ADE pairs spanning 4097 active ingredients. These data were mined from 51,460 structured product labels released in the United States, Europe, the United Kingdom, and Japan as of April 2025, using a PubMedBERT model fine‐tuned for ADE extraction. The model demonstrates state‐of‐the‐art performance (F1 = 0.90, AUROC = 0.92, AUPR = 0.95) in extracting ADEs from the “Adverse Reactions” section of product labels. The OnSIDES database is updated quarterly and standardized using RxNorm and the Medical Dictionary for Regulatory Activities (MedDRA). To systematically identify drugs with an on‐label risk of myopathy, we queried for entries mapped to the MedDRA Preferred Term “Myopathy” (code: 10028641), resulting in a final set of 59 unique drugs for subsequent analysis. A complete list of drugs associated with an elevated risk of myopathy is available in Table S2.

### 2.3 Covariates

Potential factors associated with general ADE and myopathy were identified and defined. These factors were categorized into three groups: patient‐related, drug‐related, and disease‐related risk factors. Patient‐related factors included age, gender, race, and CYP2C19/CYP3A4 phenotypes. The Association for Molecular Pathology Pharmacogenetics (PGx) Working Group publishes guidelines that summarize the must‐test PGx alleles for each gene, aiming to promote standardization of PGx gene and allele testing across clinical laboratories [21, 35]. Based on the minimum set of alleles for testing (Tier 1), the genotyping of CYP2C19 included CYP2C19*2 (c.681G>A; rs4244285), CYP2C19*3 (c.636G>A; rs4986893), and CYP2C19*17 (g.‐806C>T; rs12248560) variants [36]. The CYP2C19*2 and CYP2C19*3 alleles are associated with loss of function, while the CYP2C19*17 allele is associated with gain of function. Metabolic phenotypes based on CYP2C19 variants were categorized as follows: EM had the wild‐type genotype (CYP2C19*1/*1), UM carried at least one *17 (CYP2C19*1/*17 or *17/*17), IM had one *2 or *3 (CYP2C19*1/*2 or *1/*3), and PM carried two loss‐of‐function alleles (CYP2C19*2/*2, *3/*3, or *2/*3). For CYP3A4, the CYP3A4*22 (g.15389C>T; rs35599367) variant, known for reduced enzyme activity, was genotyped [35]. In terms of metabolic phenotypes, EM had the wild‐type (CYP3A4*1/*1), IM had a heterozygous *1/*22 genotype, and PM was identified as those with the homozygous *22/*22 genotype (Table 1).

**TABLE 1:** The predicted CYP2C19 and CYP3A4 phenotypes based on their genotypes.

Gene	Phenotype	Genotype
CYP2C19	EM	*1/*1
	UM	*1/*17, *17/*17
	IM	*1/*2, *1/*3
	PM	*2/*2, *3/*3, *2/*3
CYP3A4	EM	*1/*1
	IM	*1/*22
	PM	*22/*22

Abbreviations: CYP, cytochrome P450; EM, extensive metabolizer; IM, intermediate metabolizer; PM, poor metabolizer; UM, ultrarapid metabolizer.

Drug‐related factors included the number of unique drug exposures. Disease‐related factors included accidents or injuries, acute kidney failure, acute myocardial infarction, cancer, cardiovascular disease, coma, convulsions, dehydration, dementia, depression, diabetes, elevated white blood cell count, falls, hyperlipidemia, hypertension, hypothyroidism, impaired liver and renal function, overexertion, paraplegia, pneumonia, sepsis or septic shock, and stroke [37, 38, 39, 40, 41]. The diagnosis codes corresponding to these conditions are provided in Table S3.

### 2.4 Outcomes: Myopathy Patient Identification

Myopathy cases were identified using diagnosis codes from the International Classification of Diseases (ICD)‐9 (359.4 (toxic myopathy), 359.8 (other myopathy), 710.4 (polymyositis), 728.8 (other disorders of muscle ligament and fascia), 728.9 (unspecified disorders of muscle ligament and fascia), 729.1 (myalgia and myositis, unspecified)) or ICD‐10 (G72 (other and unspecified myopathies), M33.20 (polymyositis), M60.1 (interstitial myositis), M60.8 (other myositis), M60.9 (myositis, unspecified), M62.00 (separation of muscle (nontraumatic), unspecified site), M62.10 (other rupture of muscle (nontraumatic), unspecified site), M62.40 (contracture of muscle, unspecified site), M62.81 (muscle weakness), M62.83 (muscle spasm), M62.89 (other specified disorders of muscle), M62.9 (disorder of muscle, unspecified), M79.1 (myalgia)), or by a creatine kinase level equal to or above 10 times the upper limit of normal [42, 43]. Only the first diagnosis of myopathy was included for each patient, as subsequent diagnoses may have reflected follow‐up assessments rather than new or recurrent events.

Observation periods comprised continuous omeprazole treatment followed by a washout period to account for residual drug effects post‐cessation. The washout period was set at five half‐lives of the drug, a duration sufficient to eliminate approximately 94%–97% of the drug's effects. Half‐life information was obtained from DrugBank [44].

**FIGURE 2:**
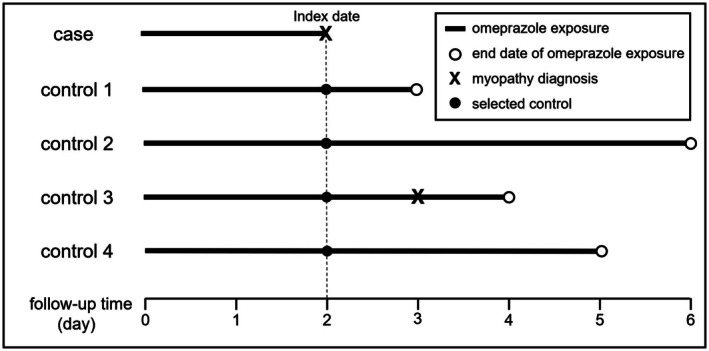
Nested case–control sampling.

We conducted a nested case–control study to investigate the association between omeprazole exposure and the risk of myopathy (Figure 2). Cases were defined as patients who received their first diagnosis of myopathy during the observation period, with the date of initial myopathy diagnosis designated as the index date. For each case, up to four controls were matched using incidence‐density sampling. Controls were selected from patients who had been exposed to omeprazole for at least the same duration as their matched case by the index date and who had not developed myopathy up to and including the index date. This approach ensured comparable exposure periods and minimized potential follow‐up bias. Sample‐size adequacy was evaluated with the powerSurvEpi R package (version 0.1.3). Assuming a conditional logistic‐regression model with one case matched to four controls, an anticipated odds ratio (OR) of 3.0, an exposure prevalence of 2%, a two‐sided *α* = 0.05, and an *R* ^2^ = 0.10 to account for modest collinearity between the exposure and covariates, the 460 matched sets available for analysis (460 cases and 1840 controls) confer an estimated 80% statistical power to detect the prespecified effect size.

Baseline characteristics of cases and controls were compared using descriptive statistics. The Shapiro–Wilk test assessed the normality of continuous variables. Normally distributed continuous data are shown as means with standard deviations, while non‐normally distributed data are shown as medians with interquartile ranges (IQR). Categorical variables were analyzed using Pearson's chi‐squared test or Fisher's exact test when expected cell counts were below 5.

### 2.5 Drug–Drug Interaction (DDI) Analysis

**FIGURE 3:**
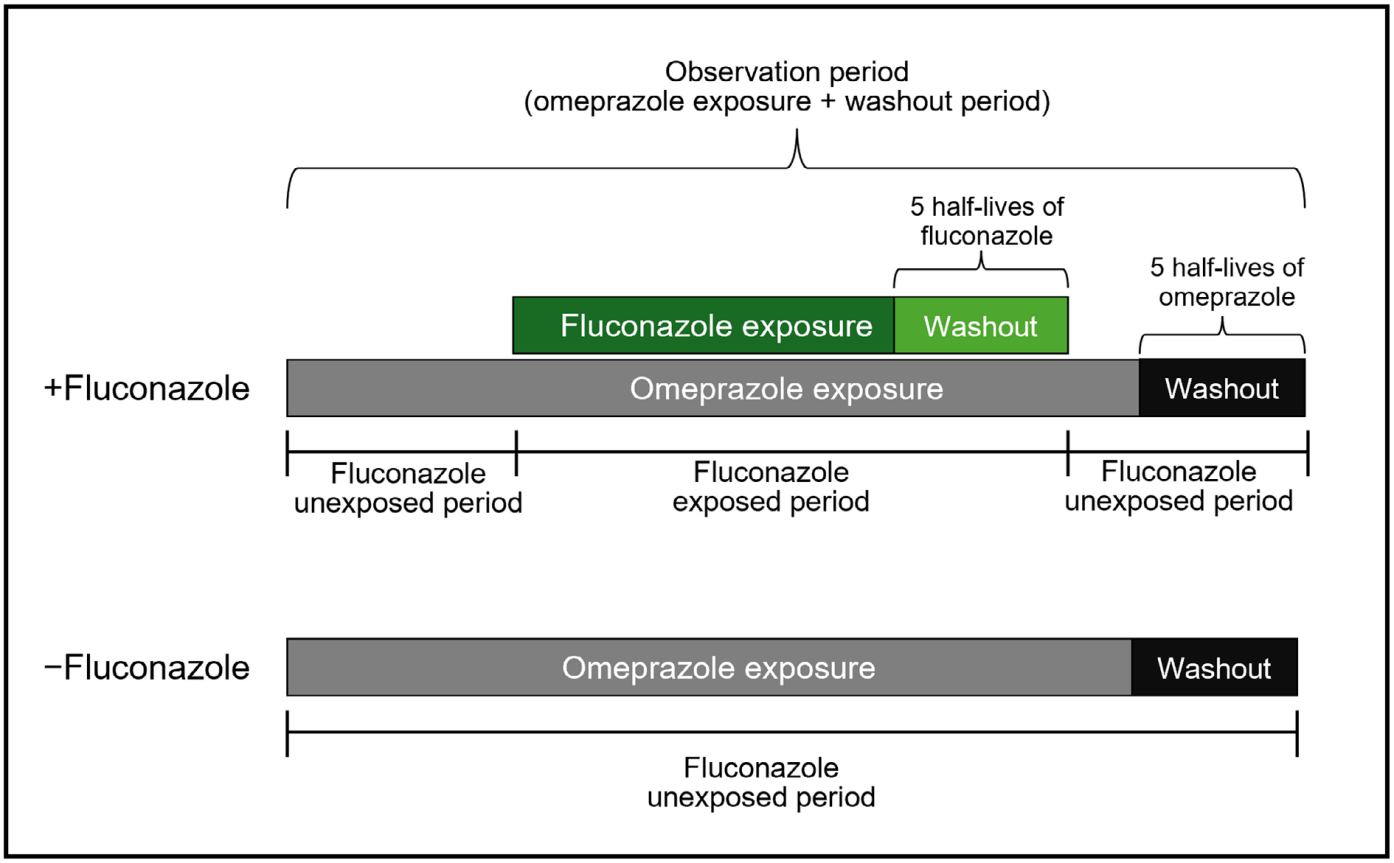
Definition of omeprazole and fluconazole exposure periods and washout windows.

For each patient, periods of overlapping exposure to both omeprazole and fluconazole during the observation were identified and designated as “fluconazole‐exposed periods,” whereas intervals of omeprazole use without fluconazole overlap were defined as “fluconazole‐unexposed periods” (Figure 3). Myopathy events occurring during fluconazole‐exposed periods were classified as DDI‐induced, whereas those during fluconazole‐unexposed periods were attributed to omeprazole alone.

We performed exact conditional logistic regression (ECLR) for univariate and multivariable analyses, stratified by matched sets, to estimate the association between concurrent fluconazole exposure and the risk of myopathy. ECLR computes the exact conditional likelihood by enumerating all possible permutations within each matched stratum, avoiding small‐sample bias and separation problems that can invalidate standard conditional logistic regression when some covariate strata have very few events [45, 46]. This regression model generated adjusted odds ratios (AORs) that approximate incidence rate ratios due to the incidence‐density sampling method. The regression model was structured as follows:

(1) $$\text{Logit}\left[P\left(\text{Omeprazole}-\text{Myopathy}=1\right)\right]=\beta_{1}\text{Fluconazole}+\beta_{2}\text{covariates}$$

Covariates with no cases or controls were excluded from both univariate and multivariable models, as their inclusion could introduce model convergence issues or result in unstable parameter estimates. The effect of overlapping fluconazole exposure among patients taking omeprazole was estimated by the term $e^{\beta_{1}}$. A statistically significant association between fluconazole exposure and increased myopathy risk was defined as an AOR greater than 1 with a *p*‐value < 0.05.

### 2.6 Drug‐Gene Interaction (DGI) Analysis

The effect of CYP2C19/CYP3A4 metabolizer phenotypes on myopathy risk was assessed with the same ECLR framework, using the CYP2C19 extensive metabolizer (EM)/CYP3A4 EM combination as reference:
(2)

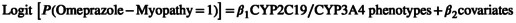




As with the DDI analysis, covariates with no cases or controls were excluded from the regression model. A DGI was considered to increase the risk of myopathy when the AOR for a CYP2C19/CYP3A4 phenotype category exceeded 1.0 and the associated *p*‐value was below 0.05.

All statistical analyses were conducted using R version 4.0.3 (R Foundation for Statistical Computing, Vienna, Austria) with the survival package [47].

## 3 Results

### 3.1 Impact of Drug–Drug Interactions on Omeprazole‐Related Myopathy

In the VUMC SD and BioVU databases, a total of 902 cases (26% male, 74% female) and 3608 controls (45% male, 55% female) were identified (Table 2). The average number of unique drug exposures (comedications) was 7 (standard deviation: 8) for cases and 5 (standard deviation: 6) for controls. The most common comorbidities among cases were hypertension, hypothyroidism, and dehydration. Elevated white blood cell count and overexertion were excluded from the regression analysis due to the absence of observed cases and controls for these variables.

**TABLE 2:** DDI: Summary of descriptive statistics summarizing patient demographic and clinical characteristics for cases and controls.

Variables	Cases *N* = 902^a^	Controls *N* = 3608^a^	*p* ^b^
Age, years	54 (15)	55 (18)	0.214
Gender			
Male	237 (26%)	1609 (45%)	< 0.001
Female	665 (74%)	1999 (55%)	
Race			
White	768 (85%)	2939 (81%)	0.551
Asian	8 (0.9%)	53 (1.5%)	
Black or African American	103 (11%)	310 (8.6%)	
Other	3 (0.3%)	6 (0.2%)	
Unknown	20 (2.2%)	300 (8.3%)	
Comedications	7 (8)	5 (6)	< 0.001
Accidents/injuries	38 (4.2%)	97 (2.7%)	0.021
AKI	110 (12%)	409 (11%)	0.484
AMI	82 (9.1%)	249 (6.9%)	0.027
Cancer	45 (5.0%)	164 (4.5%)	0.595
Cardiovascular	69 (7.6%)	205 (5.7%)	0.029
Chronic liver disease	14 (1.6%)	65 (1.8%)	0.673
Coma	4 (0.4%)	8 (0.2%)	0.274
Convulsions	47 (5.2%)	148 (4.1%)	0.144
Dehydration	170 (19%)	607 (17%)	0.153
Dementia	30 (3.3%)	45 (1.2%)	< 0.001
Depression	5 (0.6%)	11 (0.3%)	0.342
Diabetes	12 (1.3%)	19 (0.5%)	0.021
Elevated white blood cell count	0 (0%)	0 (0%)	—
Falls	1 (0.1%)	8 (0.2%)	0.698
Gout	29 (3.2%)	136 (3.8%)	0.488
Hyperlipidemia	27 (3.0%)	56 (1.6%)	0.008
Hypertension	373 (41%)	1388 (38%)	0.118
Hypothyroidism	159 (18%)	380 (11%)	< 0.001
Overexertion	0 (0%)	0 (0%)	—
Paraplegia	5 (0.6%)	4 (0.1%)	0.019
Pneumonia	109 (12%)	305 (8.5%)	< 0.001
Renal function	24 (2.7%)	58 (1.6%)	0.050
Sepsis	41 (4.5%)	168 (4.7%)	0.930
Stroke	47 (5.2%)	139 (3.9%)	0.075
CYP2C19/CYP3A4 Phenotype			
CYP2C19(EM)/CYP3A4(EM)	376 (42%)	1542 (43%)	0.334
CYP2C19(EM)/CYP3A4(IM)	39 (4.3%)	152 (4.2%)	
CYP2C19(EM)/CYP3A4(PM)	1 (0.1%)	5 (0.1%)	
CYP2C19(IM)/CYP3A4(EM)	162 (18%)	682 (19%)	
CYP2C19(IM)/CYP3A4(IM)	11 (1.2%)	56 (1.6%)	
CYP2C19(PM)/CYP3A4(EM)	39 (4.3%)	80 (2.2%)	
CYP2C19(PM)/CYP3A4(IM)	7 (0.8%)	12 (0.3%)	
CYP2C19(UM)/CYP3A4(EM)	241 (27%)	964 (27%)	
CYP2C19(UM)/CYP3A4(IM)	25 (2.8%)	112 (3.1%)	
CYP2C19(UM)/CYP3A4(PM)	1 (0.1%)	3 (< 0.1%)	
Fluconazole	46 (5.1%)	78 (2.2%)	< 0.001

Abbreviations: AKI, acute kidney injury; AMI, acute myocardial infarction; CYP, cytochrome P450; EM, extensive metabolizer; IM, intermediate metabolizer; PM, poor metabolizer; UM, ultrarapid metabolizer.

^a^ Age and comedication: Mean (standard deviation); other variables: *n* (%).

^b^ Unpaired *t*‐test or Wilcoxon rank–sum test; Fisher's exact test or Pearson's chi‐squared test.

The effect of overlapping fluconazole exposure among patients taking omeprazole had an AOR of 1.75 (95% confidence interval [CI]: 1.17–2.63; *p* = 0.007), indicating a significantly increased risk of myopathy with combined use (Table 3).

**TABLE 3:** DDI: Univariate and multivariable conditional logistic regression analysis of a nested case‐control study.

Variables	Univariate			Multivariate		
	OR	95% CI	*p*	AOR	95% CI	*p*
Age	1.00	0.99–1.00	0.271	0.99	0.99–1.00	0.016
Gender						
Male	Referent	Referent	Referent	Referent	Referent	Referent
Female	2.26	1.92–2.66	< 0.001	2.14	1.80–2.54	< 0.001
Race						
White	Referent	Referent	Referent	Referent	Referent	Referent
Asian	0.59	0.28–1.25	0.167	0.49	0.23–1.06	0.070
Black or African American	1.28	1.01–1.62	0.042	1.10	0.85–1.42	0.470
Other	1.86	0.46–7.49	0.381	1.50	0.36–6.31	0.580
Unknown	0.26	0.16–0.41	< 0.001	0.25	0.15–0.40	< 0.001
Comedications	1.03	1.02–1.04	< 0.001	1.03	1.02–1.04	< 0.001
Accidents/injuries	1.59	1.09–2.33	0.017	1.39	0.92–2.09	0.113
AKI	1.09	0.87–1.37	0.465	0.87	0.65–1.16	0.342
AMI	1.34	1.03–1.73	0.026	1.24	0.91–1.68	0.166
Cancer	1.10	0.79–1.55	0.569	1.17	0.80–1.71	0.418
Cardiovascular	1.38	1.04–1.83	0.027	0.92	0.61–1.37	0.674
Chronic liver disease	0.86	0.48–1.54	0.612	0.69	0.36–1.35	0.281
Coma	2.00	0.60–6.64	0.258	1.15	0.29–4.60	0.845
Convulsions	1.29	0.92–1.80	0.144	1.03	0.71–1.49	0.879
Dehydration	1.15	0.95–1.38	0.153	0.92	0.73–1.16	0.461
Dementia	2.77	1.72–4.45	< 0.001	1.70	0.94–3.09	0.081
Depression	1.82	0.63–5.23	0.268	1.19	0.37–3.87	0.769
Diabetes	2.53	1.23–5.20	0.012	1.99	0.88–4.46	0.096
Falls	0.50	0.06–4.00	0.513	0.49	0.06–4.14	0.512
Gout	0.85	0.57–1.27	0.430	1.02	0.65–1.59	0.934
Hyperlipidemia	1.97	1.23–3.14	0.005	1.29	0.70–2.36	0.412
Hypertension	1.13	0.97–1.31	0.113	0.95	0.79–1.14	0.581
Hypothyroidism	1.83	1.49–2.24	< 0.001	1.52	1.22–1.90	< 0.001
Paraplegia	5.00	1.34–18.6	0.016	4.96	1.25–19.7	0.023
Pneumonia	1.48	1.17–1.86	0.001	1.35	1.03–1.77	0.030
Renal function	1.69	1.04–2.76	0.034	1.19	0.62–2.27	0.598
Sepsis	0.97	0.69–1.38	0.887	0.75	0.49–1.15	0.187
Stroke	1.37	0.98–1.92	0.068	1.12	0.77–1.64	0.548
CYP2C19/CYP3A4 genotype						
CYP2C19(EM)/CYP3A4(EM)	Referent	Referent	Referent	Referent	Referent	Referent
CYP2C19(EM)/CYP3A4(IM)	1.05	0.72–1.53	0.794	0.92	0.62–1.37	0.694
CYP2C19(EM)/CYP3A4(PM)	0.80	0.09–6.89	0.842	1.00	0.12–8.69	0.998
CYP2C19(IM)/CYP3A4(EM)	0.97	0.79–1.19	0.794	0.94	0.76–1.17	0.598
CYP2C19(IM)/CYP3A4(IM)	0.80	0.42–1.55	0.511	0.58	0.29–1.16	0.123
CYP2C19(PM)/CYP3A4(EM)	1.98	1.33–2.95	0.001	1.67	1.08–2.57	0.022
CYP2C19(PM)/CYP3A4(IM)	2.33	0.92–5.94	0.076	4.07	1.49–11.1	0.006
CYP2C19(UM)/CYP3A4(EM)	1.02	0.85–1.22	0.824	1.02	0.85–1.24	0.817
CYP2C19(UM)/CYP3A4(IM)	0.92	0.59–1.44	0.724	0.88	0.55–1.42	0.611
CYP2C19(UM)/CYP3A4(PM)	1.34	0.14–12.9	0.802	1.70	0.16–17.5	0.657
Fluconazole	2.44	1.68–3.54	< 0.001	1.75	1.17–2.63	0.007

Abbreviations: 95% CI, 95% confidence interval; AKI, acute kidney injury; AMI, acute myocardial infarction; AOR, adjusted odds ratio; CYP, cytochrome P450; EM, extensive metabolizer; IM, intermediate metabolizer; OR, odds ratio; PM, poor metabolizer; UM, ultrarapid metabolizer.

### 3.2 Impact of CYP2C19 and CYP3A4 Polymorphisms in Omeprazole‐Related Myopathy

In the VUMC SD and BioVU database, a total of 862 cases (27% male, 73% female) and 3448 controls (45% male, 55% female) were identified (Table 4). The average number of unique drug exposures (comedications) was 6 (standard deviation: 8) for cases and 5 (standard deviation: 6) for controls. The most common comorbidities among cases and controls were hypertension, hypothyroidism, and dehydration. The combination genotype CYP2C19 EM/CYP3A4 EM was the most prevalent (cases: 42%, controls: 43%). As in the DDI analyses, elevated white blood cell count and overexertion were not included in the regression analysis, as they were not observed in either cases or controls.

**TABLE 4:** DGI: Summary of descriptive statistics summarizing patient demographic and clinical characteristics for cases and controls.

Variables	Cases *N* = 862^a^	Controls *N* = 3448^a^	*p* ^b^
Age, years	55 (15)	55 (18)	0.483
Gender			
Male	234 (27%)	1557 (45%)	< 0.001
Female	628 (73%)	1891 (55%)	
Race			
White	737 (85%)	2812 (82%)	0.472
Asian	8 (0.9%)	48 (1.4%)	
Black or African American	95 (11%)	292 (8.5%)	
Other	3 (0.3%)	6 (0.2%)	
Unknown	19 (2.2%)	290 (8.4%)	
Comedications	6 (8)	5 (6)	< 0.001
Accidents/injuries	36 (4.2%)	88 (2.6%)	0.016
AKI	101 (12%)	381 (11%)	0.587
AMI	79 (9.2%)	234 (6.8%)	0.019
Cardiovascular	64 (7.4%)	185 (5.4%)	0.022
Cancer	41 (4.8%)	153 (4.4%)	0.713
Chronic liver disease	13 (1.5%)	57 (1.7%)	0.881
Coma	4 (0.5%)	4 (0.1%)	0.056
Convulsions	46 (5.3%)	139 (4.0%)	0.092
Dehydration	157 (18%)	555 (16%)	0.137
Dementia	25 (2.9%)	36 (1.0%)	< 0.001
Depression	4 (0.5%)	11 (0.3%)	0.518
Diabetes	11 (1.3%)	16 (0.5%)	0.013
Elevated white blood cell count	0 (0%)	0 (0%)	—
Falls	1 (0.1%)	8 (0.2%)	0.698
Gout	28 (3.2%)	128 (3.7%)	0.610
Hyperlipidemia	25 (2.9%)	50 (1.5%)	0.005
Hypertension	357 (41%)	1298 (38%)	0.046
Hypothyroidism	148 (17%)	359 (10%)	< 0.001
Overexertion	0 (0%)	0 (0%)	—
Paraplegia	5 (0.6%)	4 (0.1%)	0.019
Pneumonia	104 (12%)	283 (8.2%)	< 0.001
Renal function	22 (2.6%)	51 (1.5%)	0.038
Sepsis	38 (4.4%)	148 (4.3%)	0.852
Stroke	45 (5.2%)	133 (3.9%)	0.084
CYP2C19/CYP3A4 genotype			
CYP2C19(EM)/CYP3A4(EM)	358 (42%)	1480 (43%)	0.199
CYP2C19(EM)/CYP3A4(IM)	37 (4.3%)	148 (4.3%)	
CYP2C19(EM)/CYP3A4(PM)	1 (0.1%)	5 (0.1%)	
CYP2C19(IM)/CYP3A4(EM)	154 (18%)	646 (19%)	
CYP2C19(IM)/CYP3A4(IM)	11 (1.3%)	48 (1.4%)	
CYP2C19(PM)/CYP3A4(EM)	35 (4.1%)	79 (2.3%)	
CYP2C19(PM)/CYP3A4(IM)	7 (0.8%)	12 (0.3%)	
CYP2C19(UM)/CYP3A4(EM)	234 (27%)	919 (27%)	
CYP2C19(UM)/CYP3A4(IM)	24 (2.8%)	108 (3.1%)	
CYP2C19(UM)/CYP3A4(PM)	1 (0.1%)	3 (< 0.1%)	

Abbreviations: AKI, acute kidney injury; AMI, acute myocardial infarction; CYP, cytochrome P450; EM, extensive metabolizer; IM, intermediate metabolizer; PM, poor metabolizer; UM, ultrarapid metabolizer.

^a^ Age and comedication: Mean (standard deviation); other variables: *n* (%).

^b^ Unpaired *t*‐test or Wilcoxon rank–sum test; Fisher's exact test or Pearson's chi‐squared test.

When evaluating the combined CYP2C19 and CYP3A4 phenotypes, individuals with the CYP2C19 PM/CYP3A4 EM phenotype had a significantly higher risk of myopathy (AOR = 1.62, 95% CI: 1.03–2.55, *p* = 0.036) compared to the reference group (CYP2C19 EM/CYP3A4 EM), whereas the CYP2C19 EM/CYP3A4 IM phenotype was not significantly associated with myopathy (Table 5). Moreover, those with the CYP2C19 PM/CYP3A4 IM phenotype exhibited an even greater risk (AOR = 4.77, 95% CI: 1.74–13.1, *p* = 0.002) than those with CYP2C19 PM/CYP3A4 EM. These findings suggest that although CYP3A4 variants on their own may not increase myopathy risk, they can intensify the risk when present alongside a CYP2C19 PM phenotype.

**TABLE 5:** DGI: Univariate and multivariable conditional logistic regression analysis of a nested case–control study.

Variables	Univariate			Multivariate		
	OR	95% CI	*p*	AOR	95% CI	*p*
Age	1.00	0.99–1.00	0.533	1.0	0.99–1.00	0.042
Gender						
Male	Referent	Referent	Referent	Referent	Referent	Referent
Female	2.20	1.87–2.60	< 0.001	2.17	1.82–2.59	< 0.001
Race						
White	Referent	Referent	Referent	Referent	Referent	Referent
Asian	0.73	0.36–1.48	0.378	0.65	0.31–1.37	0.256
Black or African American	1.24	0.97–1.59	0.083	1.11	0.85–1.46	0.428
Other	1.81	0.45–7.28	0.403	1.17	0.27–5.03	0.834
Unknown	0.25	0.16–0.40	< 0.001	0.24	0.15–0.39	< 0.001
Comedications	1.03	1.02–1.04	< 0.001	1.03	1.02–1.04	< 0.001
Accidents/injuries	1.67	1.12–2.49	0.011	1.49	0.98–2.27	0.062
AKI	1.07	0.85–1.35	0.576	0.83	0.62–1.12	0.230
AMI	1.38	1.06–1.80	0.017	1.22	0.89–1.66	0.211
Cardiovascular	1.41	1.05–1.89	0.022	0.98	0.65–1.47	0.914
Cancer	1.08	0.76–1.53	0.686	1.08	0.73–1.61	0.707
Chronic liver disease	0.91	0.50–1.67	0.764	0.80	0.41–1.56	0.513
Coma	4.00	1.00–16.0	0.050	3.10	0.68–14.1	0.144
Convulsions	1.35	0.96–1.92	0.087	1.02	0.69–1.50	0.923
Dehydration	1.16	0.96–1.41	0.134	0.98	0.77–1.24	0.859
Dementia	2.88	1.71–4.86	< 0.001	2.11	1.09–4.08	0.027
Depression	1.45	0.46–4.57	0.521	0.82	0.24–2.85	0.760
Diabetes	2.75	1.28–5.93	0.010	2.59	1.12–6.00	0.026
Falls	0.50	0.06–4.00	0.513	0.42	0.05–3.63	0.432
Gout	0.87	0.57–1.32	0.513	1.06	0.67–1.69	0.788
Hyperlipidemia	2.05	1.25–3.34	0.004	1.37	0.73–2.57	0.322
Hypertension	1.17	1.01–1.36	0.042	1.01	0.84–1.22	0.886
Hypothyroidism	1.81	1.46–2.23	< 0.001	1.46	1.17–1.83	0.001
Paraplegia	5.00	1.34–18.6	0.016	6.33	1.56–25.6	0.010
Pneumonia	1.53	1.21–1.94	< 0.001	1.36	1.02–1.80	0.034
Renal function	1.75	1.05–2.91	0.031	1.05	0.53–2.05	0.897
Sepsis	1.03	0.71–1.48	0.881	0.79	0.51–1.22	0.278
Stroke	1.37	0.97–1.95	0.073	1.03	0.70–1.52	0.880
CYP2C19/CYP3A4 genotype						
CYP2C19(EM)/CYP3A4(EM)	Referent	Referent	Referent	Referent	Referent	Referent
CYP2C19(EM)/CYP3A4(IM)	1.03	0.71–1.51	0.872	0.89	0.59–1.32	0.548
CYP2C19(EM)/CYP3A4(PM)	0.84	0.10–7.21	0.874	1.09	0.12–9.73	0.935
CYP2C19(IM)/CYP3A4(EM)	0.99	0.80–1.22	0.912	0.96	0.77–1.19	0.688
CYP2C19(IM)/CYP3A4(IM)	0.96	0.50–1.86	0.909	0.77	0.39–1.54	0.462
CYP2C19(PM)/CYP3A4(EM)	1.84	1.21–2.80	0.004	1.62	1.03–2.55	0.036
CYP2C19(PM)/CYP3A4(IM)	2.42	0.95–6.17	0.065	4.77	1.74–13.1	0.002
CYP2C19(UM)/CYP3A4(EM)	1.06	0.88–1.27	0.564	1.06	0.87–1.28	0.581
CYP2C19(UM)/CYP3A4(IM)	0.92	0.58–1.45	0.723	0.86	0.53–1.38	0.520
CYP2C19(UM)/CYP3A4(PM)	1.40	0.15–13.5	0.770	1.44	0.13–15.5	0.765

Abbreviations: 95% CI, 95% confidence interval; AKI, acute kidney injury; AMI, acute myocardial infarction; AOR, adjusted odds ratio; CYP, cytochrome P450; EM, extensive metabolizer; IM, intermediate metabolizer; OR, odds ratio; PM, poor metabolizer; UM, ultrarapid metabolizer.

## 4 Discussion

In this study, we aimed to validate the association between omeprazole‐related DDIs with fluconazole and the risk of myopathy using EHR data. Additionally, we investigated the association between CYP polymorphisms and the incidence of myopathy in patients treated with omeprazole.

Our findings indicate that even after adjusting for CYP enzyme phenotypes, co‐administration of omeprazole with fluconazole is significantly associated with an increased risk of myopathy. We hypothesize that the pharmacokinetic interaction—where metabolic inhibition leads to elevated omeprazole concentrations—may interfere with muscle cell metabolism or mitochondrial function, although further research is required to elucidate these mechanisms [4, 5]. The significant interaction between two drugs underscores the importance of careful monitoring and consideration when these medications are co‐prescribed.

Moreover, our analysis shows that individuals with a CYP2C19 PM phenotype in combination with either a CYP3A4 EM or IM phenotype have a significantly increased risk of developing myopathy [22]. Notably, among CYP2C19 PM patients, those with a CYP3A4 IM phenotype exhibit a higher OR for myopathy than those with a CYP3A4 EM phenotype. In contrast, when CYP2C19 is not in the PM category (i.e., in EM, IM, or UM), the CYP3A4 phenotype does not significantly influence myopathy risk. These results suggest that while CYP2C19 plays a primary role in omeprazole metabolism, concomitant CYP3A4 polymorphisms can further elevate myopathy risk in patients with impaired CYP2C19 function. The lack of a significant association between CYP3A4 polymorphisms and myopathy risk when considered along with CYP2C19 EM or IM may be due to the relatively smaller impact of CYP3A4 genetic variability on omeprazole metabolism compared to CYP2C19 [48]. These findings highlight a potential risk factor that clinicians should be aware of, especially in patients who have unexplained myopathic symptoms. Pharmacogenetic testing for CYP2C19 variants may help identify patients at higher risk of omeprazole‐related myopathy, aligning with existing guidelines that recommend considering the CYP2C19 phenotype in the clinical use of drugs metabolized by this enzyme [49]. For patients identified as CYP2C19 PM, it may be preferable to use alternative PPIs not extensively metabolized by CYP2C19, such as rabeprazole or pantoprazole [50], and further evaluation of the CYP3A4 phenotype in these patients could enhance risk stratification.

There are several limitations to our study. First, as a retrospective analysis, there remains potential for residual confounding and misclassification bias, which may arise from incomplete or inaccurate medical records. Our reliance on ICD codes and laboratory data for identifying myopathy cases may have led to missing cases that did not meet these criteria or were coded differently. Additionally, although our outcome definition was adapted from prior literature on drug‐induced myopathy, this definition itself has not undergone formal validation. Consequently, distinguishing myopathy specifically attributable to DDIs or genetic predispositions from other etiologies based solely on administrative coding and laboratory findings remains challenging. Second, the external validity of our results may be limited, given that the study population was drawn from a single academic medical center, potentially restricting generalizability to other patient populations or clinical settings. Third, despite adjusting for various confounders, the high rate of missing data prevented us from accounting for other potentially important factors–such as drug dosage and smoking status–that are known risk factors in DDI and DGI analyses. Lastly, we note that the BioVU AGD WGS dataset used in our study is primarily composed of individuals of Caucasian descent (83%). Although this demographic focus offers valuable insights into a Caucasian population, reliance on a single racial group in genomics research can restrict the broader applicability of our conclusions to other racial and ethnic backgrounds.

In summary, our study confirms an omeprazole–fluconazole interaction that raises myopathy risk and reveals a DGI involving CYP2C19/CYP3A4 phenotypes. These findings enhance understanding of omeprazole's safety profile and illustrate how EHR and genetic data can generate insights into adverse drug events. Although these findings alone do not warrant immediate clinical action, they establish a basis for validation in diverse prospective cohorts, which may allow accurate identification of high‐risk patients and guide individualized risk‐reduction strategies.

## Author Contributions

**Eugene Jeong:** conceptualization, investigation, funding acquisition, writing – original draft, writing – review and editing, visualization, validation, methodology, software, formal analysis, project administration, resources, data curation, supervision. **Aditi Shendre:** supervision, conceptualization, investigation, writing – review and editing, methodology, validation, formal analysis. **Yu Su:** writing – review and editing, validation, methodology, conceptualization, investigation, supervision, formal analysis. **Xingyi Guo:** resources, supervision, conceptualization, investigation, methodology, validation, writing – review and editing, formal analysis. **Lang Li:** funding acquisition, investigation, conceptualization, writing – original draft, validation, methodology, formal analysis, project administration, supervision, writing – review and editing, resources. **You Chen:** conceptualization, investigation, funding acquisition, writing – original draft, methodology, validation, writing – review and editing, formal analysis, project administration, supervision, resources.

## Conflicts of Interest

The authors declare no conflicts of interest.

## Supporting information


**Table S1:** CYP2C19 and CYP3A4 inhibitors identified from the FDA Drug.
**Table S2:** Drugs associated with an increased risk of myopathy.
**Table S3:** Diagnosis codes used to define disease‐related covariates.

## Data Availability

The VUMC SD and BioVU datasets are not publicly available.
