# Supplementary material for: Association of Omeprazole‐Related Myopathy With Drug–Drug and Drug–Gene Interactions Involving CYP2C19 and CYP3A4: A Nested Case–Control Study
Source: Pharmacotherapy. 2025 Sep 8;45(10):654–66. doi: 10.1002/phar.70058 (PMC12530008; doi:10.1002/phar.70058)
Supplement: Supplementary file 1 — Table S1: CYP2C19 and CYP3A4 inhibitors identified from the FDA Drug. Table S2: Drugs associated with an increased risk of myopathy. Table S3: Diagnosis codes used to define disease‐related covariates. [file PHAR-45-654-s001.pdf]

Supplementary Table 1. CYP2C19 and CYP3A4 inhibitors identified from the FDA Drug Development and Interaction tables and the Flockhart table.

Drug	Enzyme	RxCUI	Source
fluconazole	CYP2C19 (strong)	4450	Both
fluoxetine	CYP2C19 (strong)	4493	Both
fluvoxamine	CYP2C19 (strong)	42355	Both
ticlopidine	CYP2C19 (strong)	10594	Both
cenobamate	CYP2C19 (moderate)	2265690	FDA
esomeprazole	CYP2C19 (moderate)	283742	Flockhart
felbamate	CYP2C19 (moderate)	24812	Both
ketoconazole	CYP2C19 (moderate)	6135	Flockhart
voriconazole	CYP2C19 (moderate)	121243	Both
armodafinil	CYP2C19 (weak)	641465	Flockhart
chloramphenicol	CYP2C19 (weak)	2348	Flockhart
cimetidine	CYP2C19 (weak)	2541	Flockhart
citalopram	CYP2C19 (weak)	2556	Flockhart
isoniazid	CYP2C19 (weak)	6038	Flockhart
luliconazole	CYP2C19 (weak)	1482680	Flockhart
modafinil	CYP2C19 (weak)	30125	Flockhart
omeprazole	CYP2C19 (weak)	7646	Both
rucaparib	CYP2C19 (weak)	1862579	Flockhart
asabuvir / ombitasvir / paritaprevir / ritonavir	CYP3A (strong)	1600259	FDA
boceprevir	CYP3A (strong)	1102129	Flockhart
ceritinib	CYP3A (strong)	1535457	Both
clarithromycin	CYP3A (strong)	21212	Both
cobicistat	CYP3A (strong)	1306284	FDA
delaviridine	CYP3A (strong)	83816	Flockhart
idelalisib	CYP3A (strong)	1544460	Both
indinavir	CYP3A (strong)	114289	Both
itraconazole	CYP3A (strong)	28031	Both
ketoconazole	CYP3A (strong)	6135	Both
lopinavir / ritonavir	CYP3A (strong)	284640	FDA
mibefradil	CYP3A (strong)	83213	Flockhart
nefazodone	CYP3A (strong)	31565	Both
nelfinavir	CYP3A (strong)	134527	Both
posaconazole	CYP3A (strong)	282446	FDA
ribociclib	CYP3A (strong)	1873916	Flockhart
ritonavir	CYP3A (strong)	85762	Both
saquinavir	CYP3A (strong)	83395	Both
telaprevir	CYP3A (strong)	1102261	Flockhart
telithromycin	CYP3A (strong)	274786	Both

tucatinib	CYP3A (strong)	2361285	Flockhart
voriconazole	CYP3A (strong)	121243	Both
aprepitant	CYP3A (moderate)	358255	Both
ciprofloxacin	CYP3A (moderate)	2551	Both
conivaptan	CYP3A (moderate)	302285	FDA
crizotinib	CYP3A (moderate)	1148495	Both
diltiazem	CYP3A (moderate)	3443	Both
dronedarone	CYP3A (moderate)	233698	FDA
erythromycin	CYP3A (moderate)	4053	Both
fluconazole	CYP3A (moderate)	4450	Both
grapefruit juice	CYP3A (moderate)	1431224	Both
imatinib	CYP3A (moderate)	282388	Both
isavuconazole	CYP3A (moderate)	1720882	FDA
letermovir	CYP3A (moderate)	1988648	Flockhart
netupitant	CYP3A (moderate)	1552337	Flockhart
verapamil	CYP3A (moderate)	11170	Both
amiodarone	CYP3A (weak)	703	FDA
atomoxetine	CYP3A (weak)	38400	Flockhart
chlorzoxazone	CYP3A (weak)	2410	FDA
cilostazol	CYP3A (weak)	21107	FDA
cimetidine	CYP3A (weak)	2541	Both
clotrimazole	CYP3A (weak)	2623	FDA
cyclosporine	CYP3A (weak)	3008	FDA
entrectinib	CYP3A (weak)	2197862	Flockhart
esomeprazole	CYP3A (weak)	283742	Flockhart
fluvoxamine	CYP3A (weak)	42355	FDA
fosaprepitant	CYP3A (weak)	1731071	FDA
istradefylline	CYP3A (weak)	2199015	FDA
ivacaftor	CYP3A (weak)	1243041	Both
lesinurad	CYP3A (weak)	1731031	Flockhart
lomitapide	CYP3A (weak)	1364479	FDA
mifepristone	CYP3A (weak)	6964	Flockhart
omeprazole	CYP3A (weak)	7646	Flockhart
quercetin	CYP3A (weak)	9060	Flockhart
ranitidine	CYP3A (weak)	9143	FDA
ranolazine	CYP3A (weak)	35829	FDA
rucaparib	CYP3A (weak)	1862579	Flockhart
simeprevir	CYP3A (weak)	1482790	Flockhart
ticagrelor	CYP3A (weak)	1116632	FDA

Supplementary Table 2. Drugs associated with an increased risk of myopathy.

RxNorm ingredient	RxCUI	Drug Label Source
abacavir	190521	United States
abiraterone	1100072	United Kingdom
acitretin	16818	United States
allopurinol	519	United States
almotriptan	279645	United States
amlodipine	17767	United States
amoxicillin	723	United States
aripiprazole	89013	United Kingdom
atorvastatin	83367	United States
atracurium	1218	United Kingdom
bempedoic acid	2282403	European Union
betamethasone	1514	Japan
binimetinib	2049122	European Union
carmUnited Statestine	2105	United Kingdom
chlorothiazide	2396	United Kingdom
cisatracurium	319864	United Kingdom
clarithromycin	21212	United States
colchicine	2683	Japan
daptomycin	22299	United States
deflazacort	22396	United States
desloratadine	275635	United Kingdom
dexamethasone	3264	United States, United Kingdom
emtricitabine	276237	Japan
encorafenib	2049106	United States, European Union
erlotinib	337525	United States
ezetimibe	341248	Japan, United States, European Union
fenofibrate	8703	United States, European Union
fluvastatin	41127	United States
gemfibrozil	4719	United States
hydrochlorothiazide	5487	United States
hydrocortisone	5492	United Kingdom
hydroxychloroquine	5521	Japan, United States, United Kingdom
lamivudine	68244	United States, United Kingdom
lansoprazole	17128	United States
lenalidomide	342369	Japan
lomitapide	1364479	United States
lovastatin	6472	United States
methyldopa	6876	United Kingdom
methylprednisolone	6902	United Kingdom
nabumetone	31448	United Kingdom

ofloxacin	7623	United Kingdom
paroxetine	32937	United States
pitavastatin	861634	Japan, United States
pravastatin	42463	Japan, United States
prednisolone	8638	Japan, United Kingdom
quinapril	35208	United States
raltegravir	719872	United States, United Kingdom, European Union
rifampin	9384	United Kingdom
ritonavir	85762	United States
rocuronium	68139	United States, United Kingdom
rosuvastatin	301542	United States, United Kingdom
simvastatin	36567	Japan, United States, European Union
sonidegib	1659191	European Union
sulfamethoxazole	10180	United Kingdom
sunitinib	357977	United Kingdom
temozolomide	37776	United Kingdom, European Union
tenofovir disoproxil	300195	Japan
vamorolone	2669799	United States
zidovudine	11413	United States, United Kingdom

Supplementary Table 3. Diagnosis codes used to define disease-related covariates.

Comorbidity	ICD-9	ICD-10
Accidents/injuries	E800 -E848, E900 - E909, E910 - E915, E916 -E926, E928 - E929, E980 -E989	V00-Y99 excluding W00-19
Acute kidney injury	584.9, V56	N17.0-N17.2, N17.8-N17.9
Acute myocardial infarction	410, 411, 413	I20, I21, I22, I25
Cancer	140-209	C[0-9][0-7]
Cardiovascular diseases	41[0-4], 43[0-8], 42[5,7-8]	I0[0-2,5-9].X, I1[0-5].X, I2[0-8].X, I3[0-9].X, I4[0-9].X, I5[0-2].X, I6[0-9].X, I7[0-9].X
Chronic liver diseases	155, 571.[0-3, 4-9], 570, 57[2-3]	B18.X, I8[5-6].X, I98.X, K7[0-7].X
Coma	780.01	R40.20
Convulsions	780.31, 780.39, 345.10-345.91	R56.00, R56.9, G40.309, G40.401, G40.409, G40.311, G40.319, G40.411, G40.419, G40.A01, G40.A09, G40.A11, G40.A19, G40.301, G40.201, G40.209, G40.211, G40.219, G40.001, G40.009, G40.101, G40.109, G40.011, G40.019, G40.111, G40.119, G40.821, G40.822, G40.823, G40.824, G40.501, G40.509, G40.801, G40.802, G40.811, G40.812, G40.089, G40.B01, G40.B09, G40.803, G40.804, G40.813, G40.814, G40.89, G40.B11, G40.B19, G40.901, G40.911, G40.919
Dehydration	276.5, 276.0, 276.1	E87.0, E87.1, E87.6, E86.0
Dementia	290.X, 291.2, 292.82, 294.[1,2,8], 331.0	F0[0-3], F05.X, G3[0-1]
Depression	296.22, 296.3[0-3,9], 311	F3[0-3]
Diabetes	250.X[0-3], 357.2, 362.0X, 366.41, 648.0X	E1[0-4]
Elevated white blood cell count	288.6	D72.82X
Falls	E880 -E888	
Gout	274	M10
Hyperlipidemia	272.[0-2,4]	E78.X
Hypertension	401,402,403,404,405	I10, I11, I12, I13, I14, I15, I16

Hypothyroidism	243, 244.X	E00.0, E00.1, E00.2, E00.9, E03.0, E03.1, E89.0, E03.2, E01.8, E03.3, E03.8, E03.9
Overexertion	E927.X	X50.0
Paraplegia	344.1	G04.1, G82.20, G82.21, G82.22
Pneumonia	480.X-486.X	J120, J121, J122, J1281, J123, J1289, J129, J13, J181, J150, J151, J14, J154, J153, J1520, J15211, J15212, J1529, J158, J155, J156, A481, J158, J159, J157, J160, J168, B250, A3701, A3711, A3781, A3791, A221, B440, J17, B7781, J180, J188, J189
Renal function	016.0, 095.4, 189.[0,9], 223.0, 236.91, 250.4, 271.4, 274.1, 283.11	I1[2-3].X, N1[7-9].X
Sepsis/septic shock	38	A41.2, A41.01, A41.02, A41.11, A41.4, A41.50, A41.3, A41.51, A41.52, A41.53, A41.59, A41.89, A47.27, A41.9 R65.20 - R65.21
Stroke	430.X, 431.X, 432.1, 432.9, 992.0	I60.X, I61.X, I62.X, I163.X, T67.0XXA
